# Curcumin Alleviates Oxygen-Glucose-Deprivation/Reperfusion-Induced Oxidative Damage by Regulating miR-1287-5p/LONP2 Axis in SH-SY5Y Cells

**DOI:** 10.1155/2021/5548706

**Published:** 2021-09-18

**Authors:** Teng Zhang, Xiaomin Chen, Yueqing Qu, Yanbing Ding

**Affiliations:** ^1^Department of Traditional Chinese Medicine Encephalopathy, Hubei Provincial Hospital Traditional Chinese Medicine, Wuhan 430074, China; ^2^Department of Traditional Chinese Medicine Encephalopathy, Hubei Province Traditional Chinese Medicine Research Institute, Wuhan 430074, China

## Abstract

Oxidative stress-induced neuronal damage is a main cause of ischemia/reperfusion injury. Curcumin (Cur), the principal constituent extracted from dried rhizomes of Curcuma longa L. (turmeric), exhibits excellent antioxidant effects. Previous studies have indicated that miR-1287-5p was downregulated in patients with ischemic stroke. Additionally, we predicted that Lon Peptidase 2, Peroxisomal (LONP2), which is involved in oxidative stress regulation, is targeted by miR-1287-5p. The aim of the current study is to investigate the effect of Cur on ischemia/reperfusion damage and its underlying mechanism. To mimic ischemia/reperfusion damage environment, SH-SY5Y cells were subjected to oxygen-glucose-deprivation/reperfusion (OGD/R). OGD/R treatment downregulated miR-1287-5p and upregulated LONP2 in SH-SY5Y cells, but Cur alleviated OGD/R-induced oxidative damage and reversed the effect of OGD/R on the expression of miR-1287-5p and LONP2. Furthermore, we confirmed the interactive relationship between miR-1287-5p and LONP2 (negative regulation). We revealed that miR-1287-5p overexpression alleviated OGD/R-induced oxidative damage alleviation, similar to the effect of Cur. MiR-1287-5p inhibition accentuated OGD/R-induced oxidative damage in SH-SY5Y cells, which was reversed by Cur. The expression of LONP2 in OGD/R-treated SH-SY5Y cells was decreased by miR-1287-5p overexpression and increased by miR-1287-5p inhibition, and Cur counteracted the increase in LONP2 expression induced by miR-1287-5p inhibition. In conclusion, we suggest that Cur alleviates OGD/R-induced oxidative damage in SH-SY5Y cells by regulating the miR-1287-5p/LONP2 axis. The findings provide a theoretical basis for the clinical application of curcumin.

## 1. Introduction

Ischemic stroke is a condition with one of the highest morbidity and mortality rates worldwide, accounting for 80% of all stroke cases and leading to a series of complications, including cognitive impairment and epilepsy [1, 2]. Recombinant tissue-type plasminogen activator is the only effective drug for ischemic stroke therapy that has been approved by the Food and Drug Administration, but its use is limited by the risk of cerebral hemorrhaging and the narrow therapeutic window [3, 4]. Although neuroprotectants in animal models have attracted considerable attention, their translation into clinical applications is limited, showing disappointing results [5, 6]. Ischemic stroke is mainly caused by the coexistence of multiple complex factors, such as inflammation, oxidative stress, blood-brain barrier disorder, platelet activation, and neuronal cell apoptosis, ultimately leading to brain injury [7–10]. However, the specific pathological causes of brain injury remain unclear. Therefore, it is of great significance to deeply explore the molecular mechanism of ischemic stroke and provide new targets for clinical treatment.

Numerous studies have confirmed that restoring blood perfusion in brain tissue after ischemia can produce a large amount of reactive oxygen species (ROS) in a short time [11–13]. This leads to ischemia/reperfusion injury, resulting in blood-brain barrier destruction [14], inflammatory response [15], and apoptosis mediated by mitochondrial dysfunction [11]. Thus, alleviating ischemia/reperfusion injury by inhibiting ROS generation has received much attention [16, 17].

Curcumin (Cur) is the principal constituent extracted from the dried rhizomes of Curcuma longa L. (turmeric) and has shown excellent anti-inflammatory and antioxidant effects [18]. Accumulating evidence has revealed the neuroprotective effects of Cur during the development of ischemic stroke, including its regulation of neuron survival [19] and microglia/macrophage polarization [20]. In addition, Cur exerts a pharmacological role in many diseases by regulating the expression of microRNAs (miRNAs) [21, 22]. Xu et al. found that Cur regulated the expression of miR-7-5p to inhibit oxidative stress, apoptosis, and inflammatory response following cerebral ischemia/reperfusion, thereby preventing brain damage and cognitive dysfunction [23].

Previous studies have demonstrated that miR-1287-5p was downregulated in patients with ischemic stroke [24]. Additionally, we found through bioinformatics analysis that Lon Peptidase 2, Peroxisomal (LONP2), which is involved in oxidative stress regulation [25], is targeted by miR-1287-5p. However, whether Cur regulates the expression of miR-1287-5p during ischemia stroke to inhibit oxidative stress-induced neuronal injury has not been reported.

In the present study, we hypothesized that Cur inhibits ischemia/reperfusion-induced oxidative damage via mechanisms associated with the regulation of the miR-1287-5p expression. Herein, an oxygen-glucose-deprivation/reperfusion (OGD/R) model was established in vitro using SH-SY5Y cells. The mechanism underlying the effect of Cur in inhibiting neuronal apoptosis was then elucidated to provide a theoretical basis for the better clinical application of curcumin.

## 2. Materials and Methods

### 2.1. Cell Culture and Treatment

SH-SY5Y cells, supplied by Shanghai Institutes for Biological Sciences, Chinese Academy of Science, were incubated in Dulbecco's modified eagle medium (DMEM)/F12 (SH30023.01, Hyclone, UT, USA) containing 10% fetal bovine serum (FBS, 10270-106, Gibco, MD, USA) and maintained at 37°C with 5% CO_2_. To mimic an ischemia/reperfusion damage environment, SH-SY5Y cells were subjected to OGD/R. In detail, SH-SY5Y cells were cultured in glucose- and serum-free DMEM at 37°C in an atmosphere containing 90% N_2_, 5% CO_2_, and 5% O_2_ for 3 h. The cells were subsequently cultured in DMEM/F12 containing 10% FBS at 37°C in an atmosphere containing 5% CO_2_ and 95% air for 0, 6, 12, 24, 36, and 48 h. The optimal reperfusion treatment time point was determined, and the expression of miR-1287-5p and LONP2 was detected in OGD/R-induced cells.

Next, the effect of Cur on OGD/R-induced SH-SY5Y cells was evaluated. The harvested cells were divided into six groups based on treatment: control (CTRL), Cur, N-acety-L-cysteine (NAC, positive control), OGD/R, OGD/R + Cur, and OGD/R + NAC. CTRL cells were cultured in normal medium with no additional treatment. Cells in the Cur and NAC groups were cultured in normal medium containing 25 *μ*mol/l Cur (C110685, Aladdin, Beijing, China) or 10 mM NAC (A105422, Aladdin), respectively. Cells in the OGD/R group were induced by OGD/R (24 h) (refers to hypoxia for 24 h, the latter is consistent with this). Cells in the OGD/R + Cur and OGD/R + NAC groups were pretreated with Cur and NAC for 24 h, respectively, followed by OGD/R (24 h) induction. To verify whether Cur alleviates OGD/R-induced damage in SH-SY5Y cells by regulating miR-1287-5p, OGD/R-induced SH-SY5Y cells were transfected with mimics (miR-mimic) or inhibitors (miR-inhibitor) of miR-1287-5p with/without Cur (25 *μ*mol/l) treatment. Cell viability, apoptosis, ROS generation, and mitochondrial membrane potential were evaluated. In addition, the expression of miR-1287-5p and LONP2 and the interactive relationship between them were detected.

### 2.2. Cell Transfection

si-LONP2 (siRNA1, siRNA2, and siRNA3), miR-1287-5p mimics, and miR-1287-5p inhibitors were provided by Ribobio (Guangzhou, China). Scrambled sequences of si-LONP2, miR-1287-5p mimics, and miR-1287-5p inhibitors were used as negative controls (NC). Transfection was performed using Lipofectamine® RNAiMAX (13778030, Invitrogen, Carlsbad, CA, USA).

### 2.3. Cell Counting Kit-8 (CCK-8) Assay

The CCK-8 assay (Solarbio, Beijing, China) was carried out to detect cell viability. SH-SY5Y cells (3 × 10^3^ cell per well in 100 *μ*l of medium) were seeded into 96-well plates, incubated overnight at 37°C with 5% CO_2_, and subjected to different treatments. Thereafter, 10 *μ*l of CCK-8 solution was added to each well, and the cells were cultured for another 4 h at 37°C. The optical density was detected at 450 nm using a microplate reader (DMIL LED, Leica, Wetzlar, Germany).

### 2.4. Flow Cytometry

Flow cytometry (NovoCyte, ACEA Biosciences, San Diego, California, USA) was performed to assess apoptosis, ROS generation, and mitochondrial membrane potential. For apoptosis, 1 × 10^6^ of SH-SY5Y cells were centrifuged at 400 × g at 4°C for 5 min and resuspended in 200 *μ*l of phosphate-buffered saline (PBS, P1010, Solarbio). The cells were stained with 10 *μ*l of annexin V-fluorescein isothiocyanate (FITC, 556547, BD, Shanghai, China) and 10 *μ*l of propidium iodide (PI, 556547, BD, Shanghai, China) in the dark at 4°C for 30 min. Finally, the cells were subjected to flow cytometry. To detect ROS generation, 1 × 10^6^ of SH-SY5Y cells were resuspended in 1 ml of diluted DCFH-DA (10 *μ*mol/l) and cultured at 37°C with 5% CO_2_ for 20 min. After three washes with serum-free medium, the cells were resuspended in 500 *μ*l of PBS and subjected to flow cytometry. To assess mitochondrial membrane potential, 1 × 10^6^ SH-SY5Y cells were resuspended in 500 *μ*l of medium mixed with 500 *μ*l of JC-1 staining solution and maintained at 37°C with 5% CO_2_ for 20 min. The cells were centrifuged at 400 × g at 4°C for 3 min, resuspended in 1 ml of JC-1 staining solution, and centrifuged again at 400 × g at 4°C for 3 min. Finally, the cells were resuspended in 400 *μ*l of JC-1 staining solution and subjected to flow cytometry.

### 2.5. Dual Luciferase Reporter Assay

LONP2 cDNA comprising the predictive binding sites of miR-1287-5p was inserted into wild type (WT) pmirGLO-LONP2 vectors. Mutant (MT) LONP2 comprising point mutations of the miR-1287-5p seed region binding site was inserted into MT pmirGLO-LONP2 vectors. SH-SY5Y cells were cotransfected with LONP2-WT or LONP2-MT plasmids and miR-1287-5p mimics or miR-NC for 4 h using Lipofectamine 2000 (11668-027, Invitrogen, Carlsbad, CA, USA). Luciferase activity was detected by the dual luciferase reporter assay kit (RG027, Beyotime, Shanghai, China) according to the experiment protocol.

### 2.6. miR-1287-5p Target Prediction

The targets of miR-1287-5p were predicted using the miRDB (http://www.mirdb.org/), miRTarBase (http://mirtarbase.cuhk.edu.cn/php/index.php), TargetScan7 (http://www.targetscan.org/vert_70/), ENCORI (http://starbase.sysu.edu.cn/panCancer.php), and Jefferson (https://cm.jefferson.edu/) databases.

### 2.7. Quantitative Reverse Transcription Polymerase Chain Reaction (qRT-PCR)

Total RNA was extracted from SH-SY5Y cells using Trizol (Ambion, Texas, USA), reverse-transcribed into cDNA, and amplified using PCR. qRT-PCR amplification conditions are shown in Table 1. The sequences are as follows: miR-1287-5p forward, 5′-GGGTGCTGGATCAGTGG-3′, reverse, 5′-AACTGGTGTCGTGGAGTCGGC-3′; U6 (endogenous control) forward, 5′-CTCGCTTCGGCAGCACATATACT-3′, reverse, 5′-ACGCTTCACGAATTTGCGTGTC-3′. The data were analyzed using the 2^-*ΔΔ*Ct^ method [26].

### 2.8. Western Blot

Total proteins were extracted from SH-SY5Y cells. After protein content was quantified, 20 *μ*g of proteins was separated and transferred onto polyvinylidene fluoride membranes (Millipore, MA, USA). After blocking with 5% skim milk, the membranes were incubated for 1 h with primary antibodies against LONP2 (PAB32748, Bioswamp, Wuhan, China), B-cell lymphoma 2 (Bcl-2, PAB3004, Bioswamp), Bcl-2/Bcl-xl-associated death promoter (Bad, MAB37156, Bioswamp), cleaved caspase 3 (ab2302, Abcam, Cambridge, UK), cytochrome C (Cyt-c, ab13575, Abcam), and *β*-actin (PAB36265, Bioswamp). Thereafter, the membranes were incubated with goat anti-rabbit (SAB43706, Bioswamp) or anti-mouse (SAB43714, Bioswamp) IgG secondary antibodies for 1 h. *β*-Actin acted as the internal reference.

### 2.9. Statistical Analysis

Data are presented as the mean ± standard deviation (SD). Statistic difference among data was analyzed using one-way analysis of variance followed by Tukey's tests. *p* < 0.05 was considered to be statistically significant. All experiments were performed in triplicate.

## 3. Results

### 3.1. LONP2 Is Targeted by miR-1287-5p

**Figure 1:**
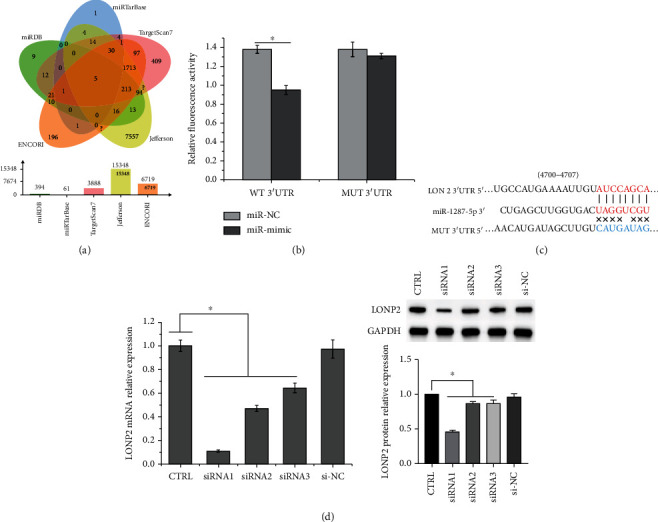
LONP2 is targeted by miR-1287-5p. (a) Targets of miR-1287-5p were predicted by miRDB, miRTarBase, TargetScan7, ENCORI, and Jefferson databases. (b) Binding sites between LONP2 and miR-1287-5p mimic. (c) Relative luciferase activity of the LONP2-WT and LONP2-MUT reporter plasmids in SH-SY5Y cells after transfection with miR-1287-5p mimics. The data represent the mean ± SD, *n* = 3. ^∗^*p* < 0.05.

The targets of miR-1287-5p were screened using the miRDB, miRTarBase, TargetScan7, ENCORI, and Jefferson databases (Supplementary 1). Among the targets, five (LONP2, RFX7, ISY1-RAB43, RAB43, and BSCL2) were identified in all five databases (Figure 1(a)). Since LONP2 was reported to be associated with oxidative stress [25], we selected LONP2 as the target for investigation. The targeting relationship between LONP2 and miR-1287-5p was confirmed using the dual luciferase reporter assay. miR-1287-5p mimics reduced the luciferase activity of WT LONP2 (Figures 1(b) and 1(c)), demonstrating that binding sites exist between miR-1287-5p and LONP2.

The expression of miR-1287-5p and LONP2 in SH-SY5Y cells was detected after transfection with si-LONP2 or miR-1287-5p mimics. Among siRNA1, 2, and 3, siRNA1 showed best effect in suppressing the mRNA expression of LONP2 and was thus selected for the subsequent experiments (Figure 1(d)).

### 3.2. OGD/R Inhibits SH-SY5Y Cell Growth, Downregulates miR-1287-5p Expression, and Upregulates LONP2 Expression

**Figure 2:**
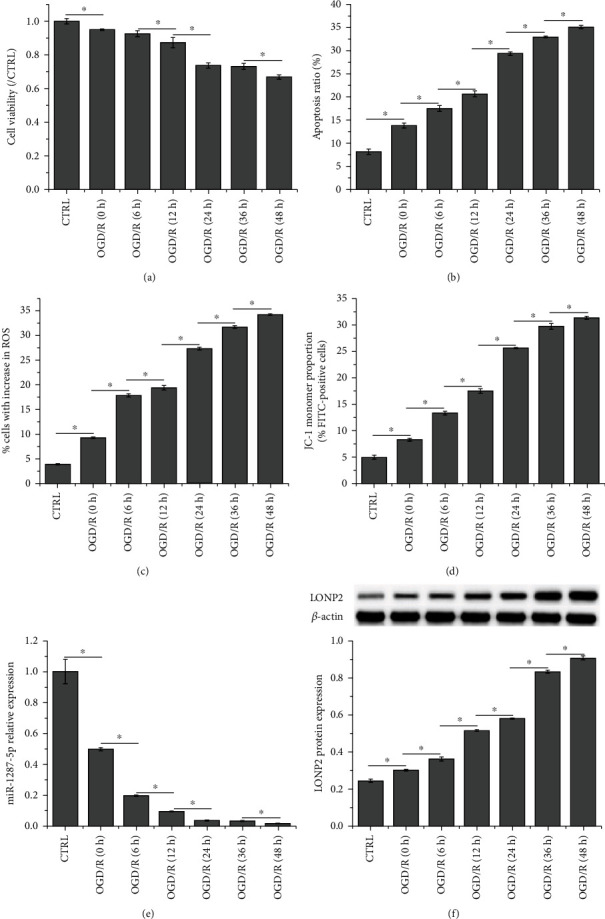
OGD/R inhibits SH-SY5Y cell growth, downregulates miR-1287-5p expression, and upregulates LONP2 expression. (a) CCK-8 assay was performed to assess the viability of OGD/R-induced SH-SY5Y cells. Flow cytometry was performed to detect (b) apoptosis, (c) ROS production, and (d) mitochondrial membrane potential in OGD/R-induced SH-SY5Y cells. (e) qRT-PCR was performed to detect miR-1287-5p expression in OGD/R-induced SH-SY5Y cells. (f) Western blot was performed to detect LONP2 expression in OGD/R-induced SH-SY5Y cells. The data represent the mean ± SD, *n* = 3. ^∗^*p* < 0.05 vs. CTRL.

As shown in Figures 2(a)–2(d) (the original results of flow cytometry are shown in Figure S1-S3), OGD/R inhibited cell viability, enhanced apoptosis and ROS generation, and induced an overall decrease in mitochondrial membrane potential in SH-SY5Y cells in a time-dependent manner. In addition, OGD/R decreased the expression of miR-1287-5p and increased that of LONP2 in a time-dependent manner (Figures 2(e) and 2(f)). As the cell viability after 24 h of reperfusion treatment was similar to that of 36 h of treatment, 24 h was selected as the time point for the subsequent experiments.

### 3.3. Cur Alleviates OGD/R-Induced SH-SY5Y Cell Damage, Upregulates miR-1287-5p Expression, and Downregulates LONP2 Expression in SH-SY5Y Cells

**Figure 3:**
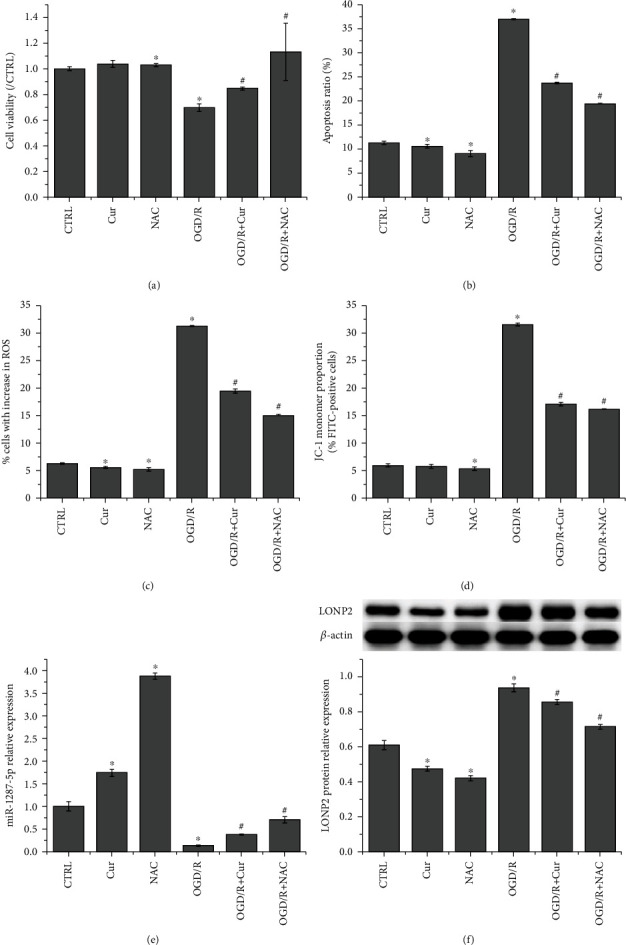
Cur alleviates OGD/R-induced SH-SY5Y cell damage, upregulates miR-1287-5p expression, and downregulates LONP2 expression in SH-SY5Y cells. (a) CCK-8 assay was performed to assess the viability of SH-SY5Y cells. Flow cytometry was performed to detect (b) apoptosis, (c) ROS production, and (d) mitochondrial membrane potential in SH-SY5Y cells. (e) qRT-PCR was performed to detect miR-1287-5p expression in SH-SY5Y cells. (f) Western blot was performed to detect LONP2 expression in SH-SY5Y cells. The data represent the mean ± SD, *n* = 3. ^∗^*p* < 0.05 vs. CTRL, ^#^*p* < 0.05 vs. OGD/R.

OGD/R-induced SH-SY5Y cells were treated with Cur and CCK-8 assay indicated that Cur or NAC (positive control) treatment recovered the viability of SH-SY5Y cells after it was suppressed by OGD/R (Figure 3(a)). Flow cytometry demonstrated that both Cur and NAC suppressed OGD/R-induced apoptosis (Figure 3(b)) and ROS production (Figure 3(c)) in SH-SY5Y cells and caused an increase in overall mitochondrial membrane potential (Figure 3(d)) (original results of flow cytometry are shown in Figure S4-S6). Additionally, Cur enhanced the expression of miR-1287-5p (Figure 3(e)) and decreased the protein expression of LONP2 in SH-SY5Y cells (Figure 3(f)), while reversing the effect of OGD/R on miR-1287-5p and LONP2 expression. These results indicated that Cur alleviates OGD/R-induced damage, upregulated miR-1287-5p expression, and downregulated LONP2 expression in SH-SY5Y cells.

### 3.4. Cur Alleviates OGD/R-Induced SH-SY5Y Cell Damage by Regulating miR-1287-5p/LONP2 Axis

**Figure 4:**
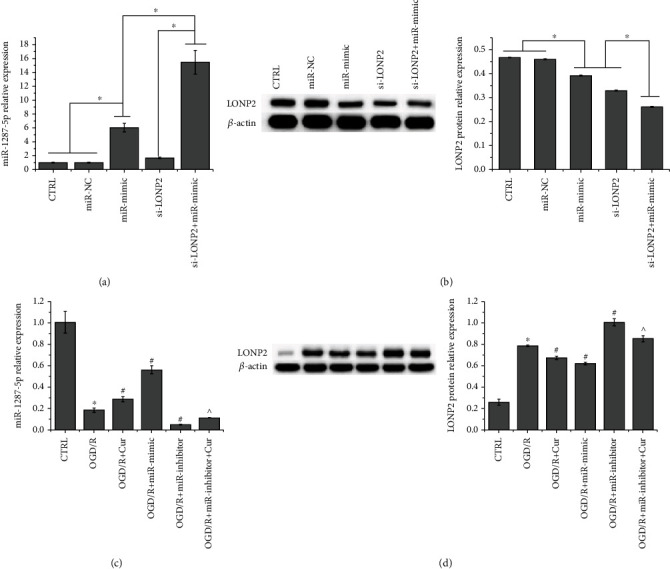
There is a reciprocal relationship between miR-1287-5p and LONP2. (a) qRT-PCR was performed to detect the mRNA expression of LONP2 in SH-SY5Y cells after siRNA transfection. (b) qRT-PCR was performed to detect miR-1287-5p expression in SH-SY5Y cells. (c) Western blot was performed to detect the protein expression of LONP2 in SH-SY5Y cells. The data represent the mean ± SD, *n* = 3. ^∗^*p* < 0.05.

**Figure 5:**
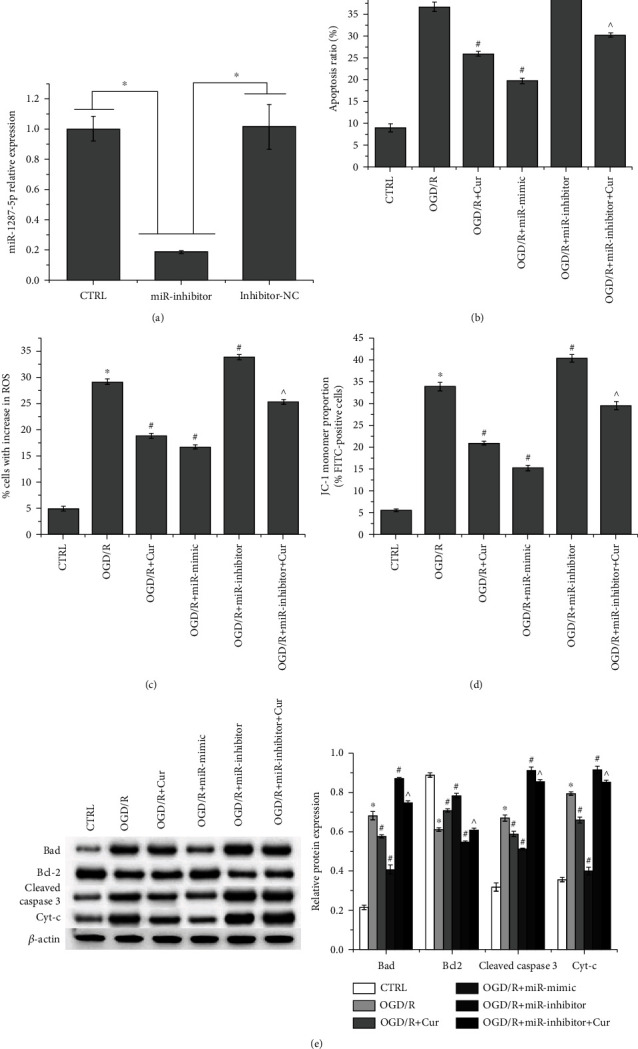
Cur alleviates OGD/R-induced SH-SY5Y cell damage by regulating miR-1287-5p/LONP2 axis. (a) qRT-PCR was performed to detect miR-1287-5p expression in SH-SY5Y cells after miR-1287-5p inhibitor transfection. Flow cytometry was performed to detect (b) apoptosis, (c) ROS production, and (d) mitochondrial membrane potential in SH-SY5Y cells. (e) Western blot was performed to detect the expression of apoptosis-related proteins in SH-SY5Y cells. The data represent the mean ± SD, *n* = 3. ^∗^*p* < 0.05 vs. CTRL, #*p* < 0.05 vs. OGD/R, ^*p* < 0.05 vs. OGD/R + miR-inhibitor.

The qRT-PCR and western blot demonstrated that both miR-1287-5p mimic and si-LONP2 increased miR-1287-5p expression and decreased that of LONP2, which further confirmed that LONP2 is a target of miR-1287-5p (Figures 4(a) and 4(b)). Both si-NC and miR-NC showed no effect. In addition, compared to nontreated OGD/R-induced cells, miR-1287-5p inhibitors downregulated the expression of miR-1287-5p (Figure 4(c)) and upregulated that of LONP2 (Figure 4(d)), but these effects were reversed by Cur. Collectively, LONP2 is verified as a target of miR-1287-5p. As shown in Figure 5(a), the miR-1287-5p inhibitor suppressed the expression of miR-1287-5p, whereas NC showed no effect on miR-1287-5p expression. Flow cytometry indicated that compared to nontreated OGD/R-induced SH-SY5Y cells, miR-1287-5p mimics decreased apoptosis (Figure 5(b)) and ROS generation (Figure 5(c)) and increased the overall mitochondrial membrane potential (Figure 5(d)) (the original results of flow cytometry are shown in Figure S7-S9). MiR-1287-5p inhibitors promoted apoptosis and ROS generation and decreased the overall mitochondrial membrane potential, but these effects were counteracted by Cur treatment. Western blot detection of apoptosis-related proteins revealed that OGD/R increased the expression of proapoptosis proteins (Bad, cleaved caspase 3, and Cyt-c) and decreased that of the antiapoptosis protein Bcl-2, but these effects were reversed by Cur (Figure 5(e)). Compared to nontreated OGD/R-induced cells, miR-1287-5p mimics decreased the expression of Bad, cleaved-caspase 3, and Cyt-c and increased that of Bcl-2, while miR-1287-5p inhibitors showed the opposite effect. The effect of miR-1287-5p inhibitors on the expression of apoptosis-related proteins was reversed by Cur. Collectively, these results indicated that Cur alleviates OGD/R-induced SH-SY5Y cell damage, and this process is possibly mediated via its regulation of the miR-1287-5p/LONP2 axis.

## 4. Discussion

The current study showed that Cur alleviated OGD/R-induced damage in SH-SY5Y cells by inhibiting apoptosis and ROS generation. In addition, Cur reversed the OGD/R-induced changes in the expression of miR-1287-5p and LONP2. We further confirmed the binding relationship between miR-1287-5p and LONP2. The effect of miR-1287-5p overexpression in alleviating OGD/R-induced oxidative damage is similar to that of Cur. miR-1287-5p inhibition accentuated the effect of OGD/R on oxidative damage in SH-SY5Y cells, which was reversed by Cur. These findings demonstrated that Cur alleviates OGD/R-induced damage in SH-SY5Y cells via possible regulation of the miR-1287-5p/LONP2 axis.

Cur exhibits a wide range of pharmacological effects including antioxidant, antiangiogenic, anti-inflammatory, and antiproliferative activities, thus showing therapeutic efficacy against human diseases such as cancer, neurological diseases, cardiovascular diseases, and Crohn's disease [27]. Studies have suggested the protective effects of Cur against ischemia/reperfusion through the regulation of various pathways. For example, Chen et al. found that Cur ameliorated ischemia/reperfusion-induced late kidney fibrosis by regulating the APPL1/Akt pathway [28]. Xu et al. showed that Cur exerted neuroprotective effects against cerebral ischemia/reperfusion injury by regulating the MEK/ERK/CREB pathway [29]. Yang et al. demonstrated that Cur suppressed myocardial ischemia/reperfusion-induced mitochondrial oxidative damage by activating SIRT1 [30]. In this work, we found that Cur exerted neuroprotective effects against ischemia/reperfusion injury *in vitro* by regulating the miR-1287-5p/LONP2 pathway. Specifically, these neuroprotective effects were validated by the Cur-mediated inhibition of ROS and apoptosis in SH-SY5Y cells.

LONP2 has been suggested to enhance oxidative stress by increasing ROS production [25], wherein mitochondria are the main source of ROS and a key target of ROS-induced damage [31, 32]. Evidence has revealed the association between mitochondrial ROS and the induction of apoptosis, suggesting that intracellular ROS production may contribute to conserved apoptotic events [33, 34]. Exposure to high levels of ROS contributed to mitochondrial apoptosis by triggering the release of Cyt-c into the cytosol [35–37], resulting in the formation of caspase 9. Then, caspase 9 promotes the proteolytic cleavage of intracellular proteins that are involved in apoptosis, such as caspase 3 [38–40]. Cyt-c release is modulated by the Bax and Bcl-2 to the outer mitochondrial membrane, which leads to the decrease in mitochondrial membrane potential [41–43]. The loss of mitochondrial membrane potential is related to mitochondrial dysfunction, which could in turn induce ROS production and initiate mitochondria-mediated apoptotic signaling [44]. The findings of this work indicated that Cur inhibited OGD/R-triggered apoptosis and ROS generation and recovered OGD/R-impaired mitochondrial membrane potential. In addition, Cur restored the OGD/R-suppressed expression of Bcl-2 and reduced the OGD/R-enhanced expression of cleaved caspase 3 and Cyt-c. Collectively, the results indicated that Cur alleviated OGD/R-induced damage by suppressing oxidative stress-induced mitochondrial apoptosis.

## 5. Conclusion

In conclusion, this work verified our hypothesis that Cur inhibits ischemia/reperfusion-induced oxidative damage, and the mechanism was related to its regulatory effect on the miR-1287-5p/LONP2 axis. This work provides a theoretical basis for the better clinical application of curcumin. Follow-up studies will be designed in *vivo* to complement the current study.

## Figures and Tables

**Table 1:** The qRT-PCR amplification condition.

95°C	3 min	
95°C	5 sec	40 cycles
56°C	10 sec	
72°C	25 sec	
Melt curve		
65°C	5 sec	0.5°C
95°C		

## Data Availability

The data used to support the findings of this study are included within the article.

## Supplementary Materials

Supplementary Materials Figure S1-S3: flow cytometry was performed to detect (S1) apoptosis, (S2) ROS production, and (S3) mitochondrial membrane potential in OGD/R-induced SH-SY5Y cells. Figure S4-S6: flow cytometry was performed to detect (S4) apoptosis, (S5) ROS production, and (S6) mitochondrial membrane potential in SH-SY5Y cells. Figure S7-S9: flow cytometry was performed to detect (S7) apoptosis, (S8) ROS production, and (S9) mitochondrial membrane potential in transfected SH-SY5Y cells.
