# Supplementary figures and images for: Curcumin Alleviates Oxygen-Glucose-Deprivation/Reperfusion-Induced Oxidative Damage by Regulating miR-1287-5p/LONP2 Axis in SH-SY5Y Cells
Source: Anal Cell Pathol (Amst). 2021 Sep 18;2021:5548706. doi: 10.1155/2021/5548706 (PMC8476263; doi:10.1155/2021/5548706)

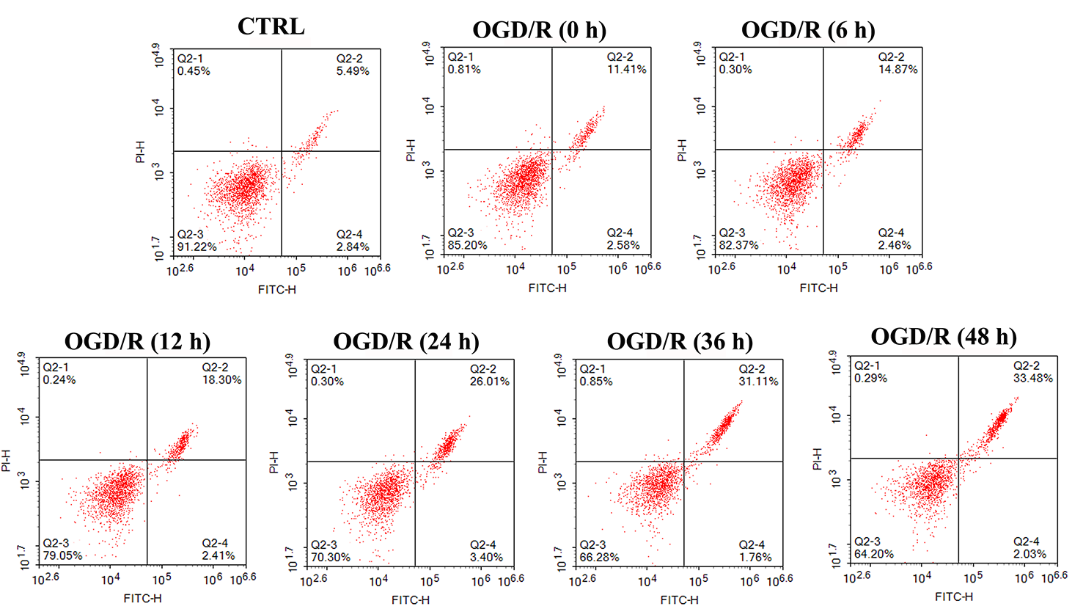

Supplement: Supplementary Materials — Figure S1-S3: flow cytometry was performed to detect (S1) apoptosis, (S2) ROS production, and (S3) mitochondrial membrane potential in OGD/R-induced SH-SY5Y cells. Figure S4-S6: flow cytometry was performed to detect (S4) apoptosis, (S5) ROS production, and (S6) mitochondrial membrane potential in SH-SY5Y cells. Figure S7-S9: flow cytometry was performed to detect (S7) apoptosis, (S8) ROS production, and (S9) mitochondrial membrane potential in transfected SH-SY5Y cells. [file 5548706.f1.zip › S1.docx]

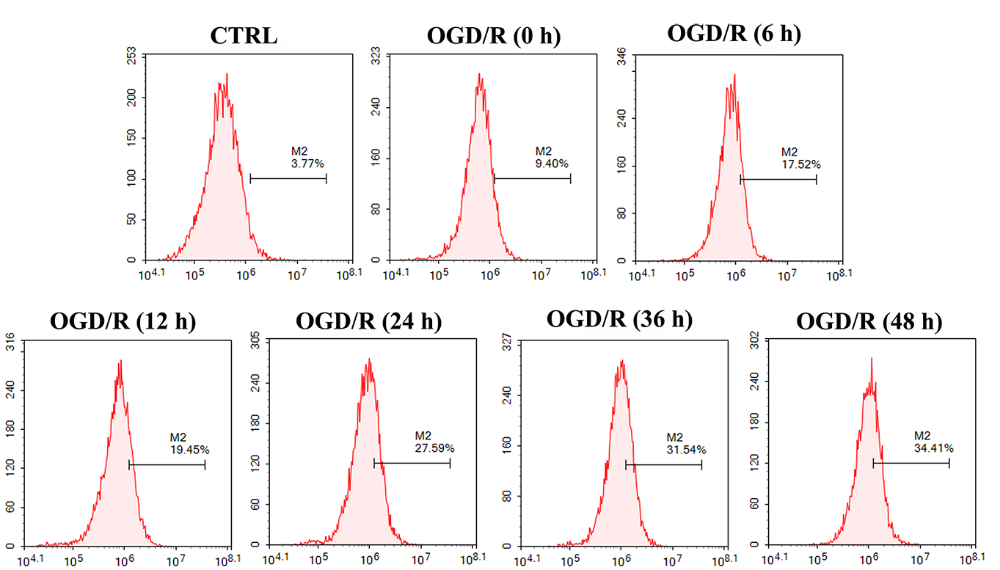

Supplement: Supplementary Materials — Figure S1-S3: flow cytometry was performed to detect (S1) apoptosis, (S2) ROS production, and (S3) mitochondrial membrane potential in OGD/R-induced SH-SY5Y cells. Figure S4-S6: flow cytometry was performed to detect (S4) apoptosis, (S5) ROS production, and (S6) mitochondrial membrane potential in SH-SY5Y cells. Figure S7-S9: flow cytometry was performed to detect (S7) apoptosis, (S8) ROS production, and (S9) mitochondrial membrane potential in transfected SH-SY5Y cells. [file 5548706.f1.zip › S2.docx]

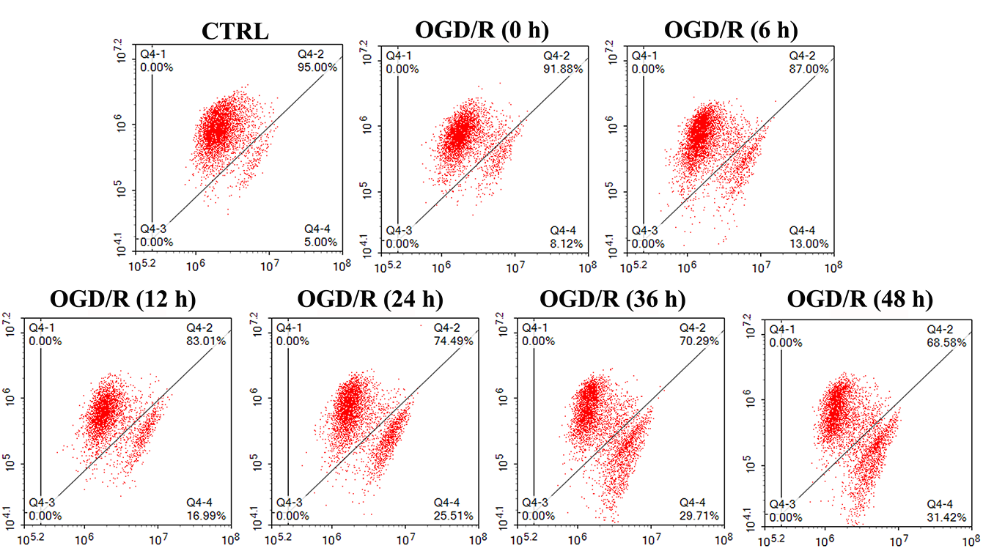

Supplement: Supplementary Materials — Figure S1-S3: flow cytometry was performed to detect (S1) apoptosis, (S2) ROS production, and (S3) mitochondrial membrane potential in OGD/R-induced SH-SY5Y cells. Figure S4-S6: flow cytometry was performed to detect (S4) apoptosis, (S5) ROS production, and (S6) mitochondrial membrane potential in SH-SY5Y cells. Figure S7-S9: flow cytometry was performed to detect (S7) apoptosis, (S8) ROS production, and (S9) mitochondrial membrane potential in transfected SH-SY5Y cells. [file 5548706.f1.zip › S3.docx]

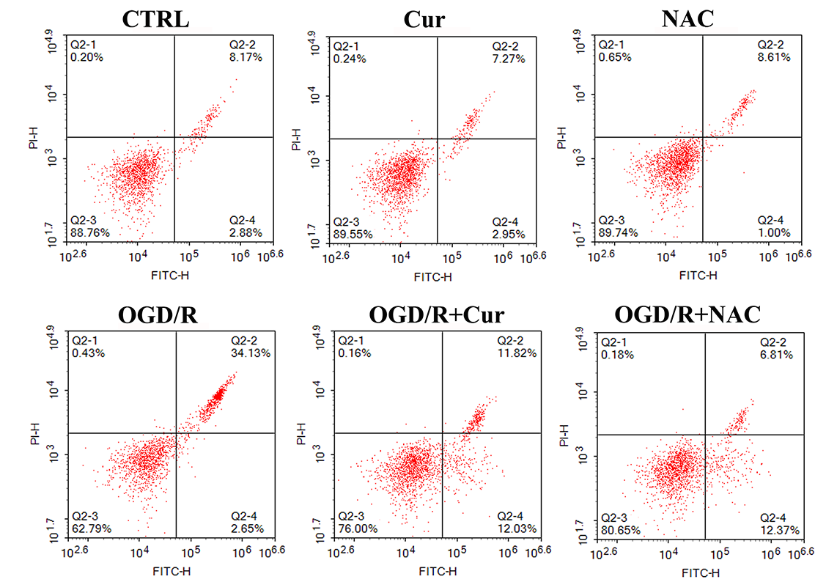

Supplement: Supplementary Materials — Figure S1-S3: flow cytometry was performed to detect (S1) apoptosis, (S2) ROS production, and (S3) mitochondrial membrane potential in OGD/R-induced SH-SY5Y cells. Figure S4-S6: flow cytometry was performed to detect (S4) apoptosis, (S5) ROS production, and (S6) mitochondrial membrane potential in SH-SY5Y cells. Figure S7-S9: flow cytometry was performed to detect (S7) apoptosis, (S8) ROS production, and (S9) mitochondrial membrane potential in transfected SH-SY5Y cells. [file 5548706.f1.zip › S4.docx]

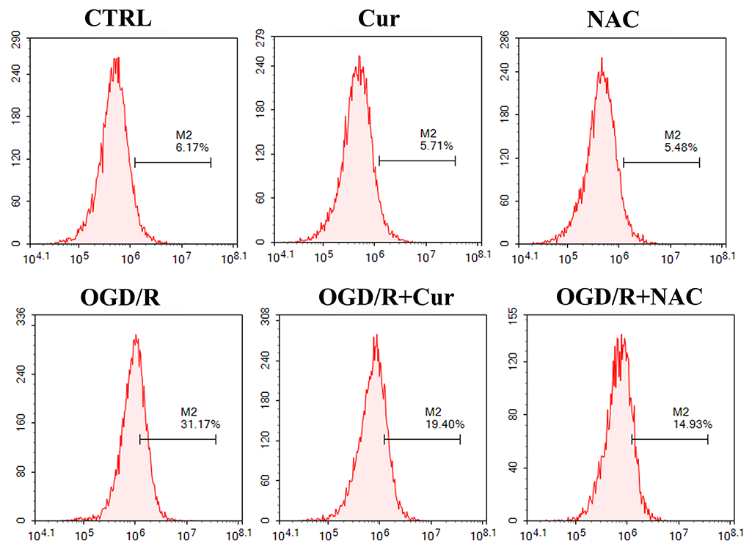

Supplement: Supplementary Materials — Figure S1-S3: flow cytometry was performed to detect (S1) apoptosis, (S2) ROS production, and (S3) mitochondrial membrane potential in OGD/R-induced SH-SY5Y cells. Figure S4-S6: flow cytometry was performed to detect (S4) apoptosis, (S5) ROS production, and (S6) mitochondrial membrane potential in SH-SY5Y cells. Figure S7-S9: flow cytometry was performed to detect (S7) apoptosis, (S8) ROS production, and (S9) mitochondrial membrane potential in transfected SH-SY5Y cells. [file 5548706.f1.zip › S5.docx]

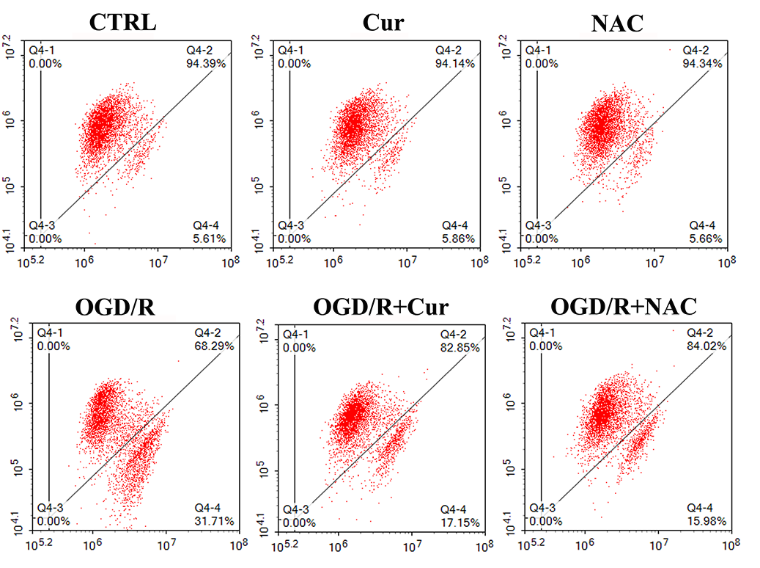

Supplement: Supplementary Materials — Figure S1-S3: flow cytometry was performed to detect (S1) apoptosis, (S2) ROS production, and (S3) mitochondrial membrane potential in OGD/R-induced SH-SY5Y cells. Figure S4-S6: flow cytometry was performed to detect (S4) apoptosis, (S5) ROS production, and (S6) mitochondrial membrane potential in SH-SY5Y cells. Figure S7-S9: flow cytometry was performed to detect (S7) apoptosis, (S8) ROS production, and (S9) mitochondrial membrane potential in transfected SH-SY5Y cells. [file 5548706.f1.zip › S6.docx]

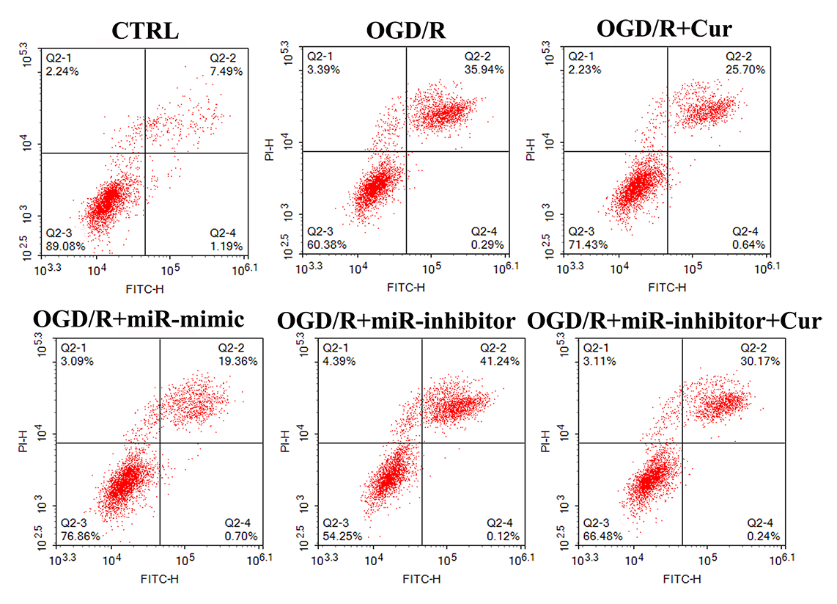

Supplement: Supplementary Materials — Figure S1-S3: flow cytometry was performed to detect (S1) apoptosis, (S2) ROS production, and (S3) mitochondrial membrane potential in OGD/R-induced SH-SY5Y cells. Figure S4-S6: flow cytometry was performed to detect (S4) apoptosis, (S5) ROS production, and (S6) mitochondrial membrane potential in SH-SY5Y cells. Figure S7-S9: flow cytometry was performed to detect (S7) apoptosis, (S8) ROS production, and (S9) mitochondrial membrane potential in transfected SH-SY5Y cells. [file 5548706.f1.zip › S7.docx]

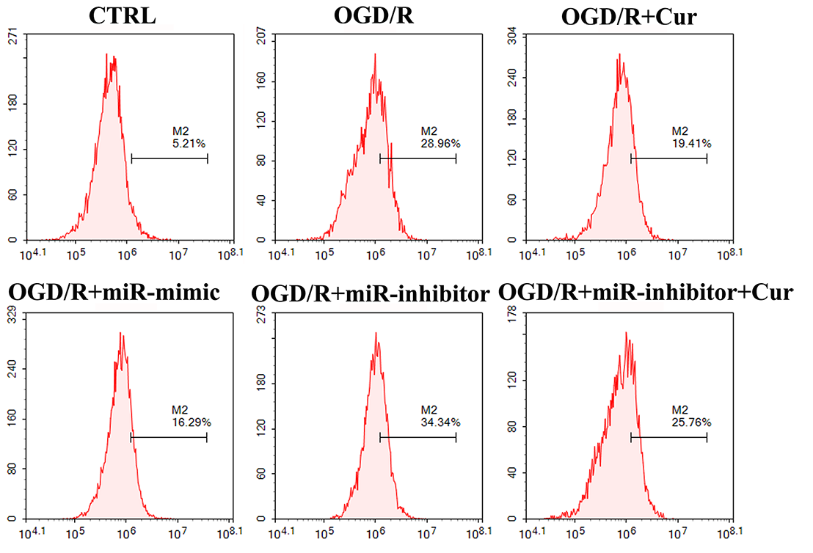

Supplement: Supplementary Materials — Figure S1-S3: flow cytometry was performed to detect (S1) apoptosis, (S2) ROS production, and (S3) mitochondrial membrane potential in OGD/R-induced SH-SY5Y cells. Figure S4-S6: flow cytometry was performed to detect (S4) apoptosis, (S5) ROS production, and (S6) mitochondrial membrane potential in SH-SY5Y cells. Figure S7-S9: flow cytometry was performed to detect (S7) apoptosis, (S8) ROS production, and (S9) mitochondrial membrane potential in transfected SH-SY5Y cells. [file 5548706.f1.zip › S8.docx]

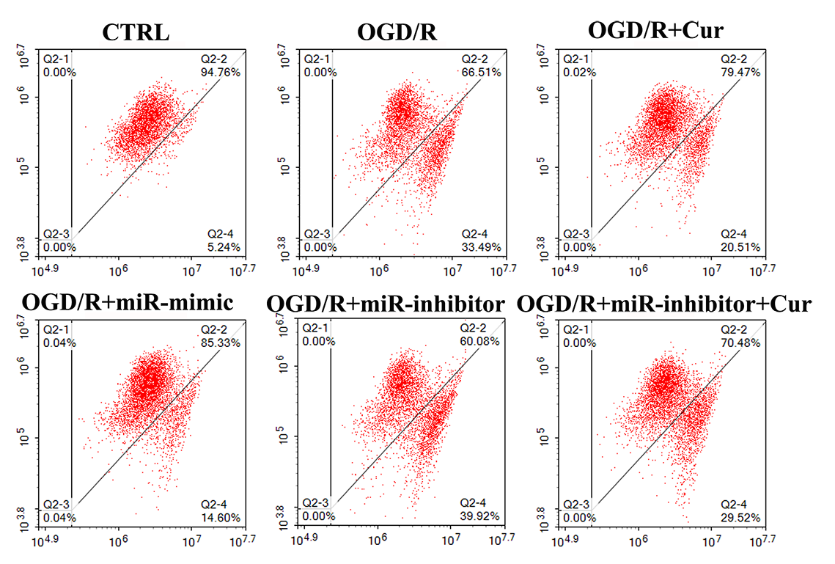

Supplement: Supplementary Materials — Figure S1-S3: flow cytometry was performed to detect (S1) apoptosis, (S2) ROS production, and (S3) mitochondrial membrane potential in OGD/R-induced SH-SY5Y cells. Figure S4-S6: flow cytometry was performed to detect (S4) apoptosis, (S5) ROS production, and (S6) mitochondrial membrane potential in SH-SY5Y cells. Figure S7-S9: flow cytometry was performed to detect (S7) apoptosis, (S8) ROS production, and (S9) mitochondrial membrane potential in transfected SH-SY5Y cells. [file 5548706.f1.zip › S9.docx]
